# Mucoepidermoid Carcinoma of Uterine Cervix: A Distinct Pathological and Clinical Entity

**DOI:** 10.1155/2015/491875

**Published:** 2015-05-10

**Authors:** Ilker Selcuk, Bulent Ozdal, Mengu Turker, Alp Usubutun, Tayfun Gungor, Mehmet Mutlu Meydanli

**Affiliations:** ^1^Gynecologic Oncology Unit, Zekai Tahir Burak Women's Health, Education and Research Hospital, Ankara, Turkey; ^2^Pathology Unit, Zekai Tahir Burak Women's Health, Education and Research Hospital, Ankara, Turkey; ^3^Department of Pathology, Hacettepe University Faculty of Medicine, Ankara, Turkey; ^4^Department of Obstetrics and Gynecology, Hitit University Faculty of Medicine, Corum, Turkey

## Abstract

Mucoepidermoid carcinoma of uterine cervix is a rare tumor that has some individual features. Defining risk factors after surgery shape the postoperative treatment modality on cervical cancer patients. Although there is not a well-known strategy for the postoperative follow-up of mucoepidermoid carcinoma, the aggressive behaviour of this tumor makes the gynecological oncologists choose liberal therapies on these patients.

## 1. Introduction

Mucoepidermoid carcinoma (MEC) is a malignant tumor that originates from epithelial tissues and produces intracellular mucin which is commonly seen in salivary glands [1]. However MEC of uterine cervix is a rare condition in which the tumor has an appearance of a squamous cell carcinoma without glandular formation and contains intracellular mucin [2]. In addition to the debate in pathological diagnosis regarding squamous or adenosquamous cervical carcinoma and MEC, MEC has some individual features that direct the gynecological oncologist on a serious clinical follow-up. Here we describe two patients of cervical cancer with a distinct pathological entity, mucoepidermoid carcinoma.

## 2. Case Report 1

**Figure 1:**
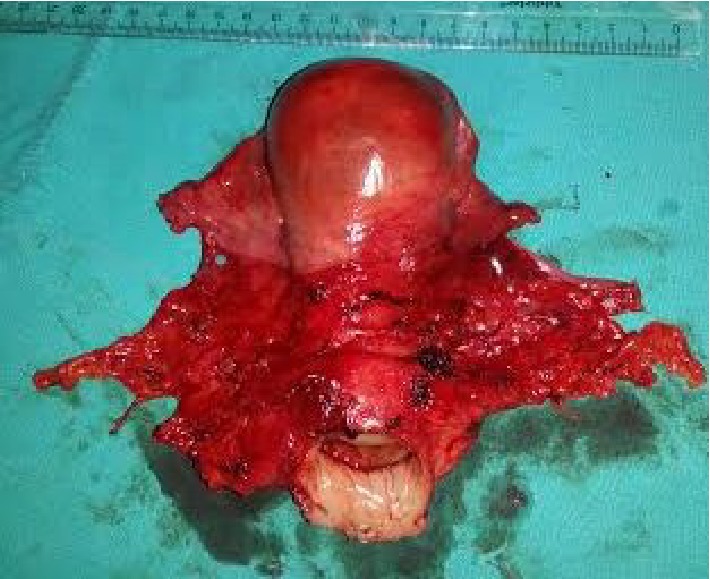
Radical hysterectomy specimen.

**Figure 2:**
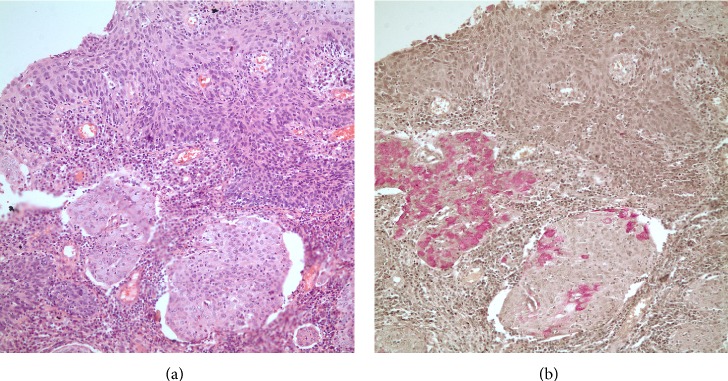
(a) Histopathologic evaluation, H&Ex40: squamous cells and intracytoplasmic mucin containing cells appearing as signet cells. (b) Histopathologic evaluation, PASx40: mucinous component does not show any glandular formation and is solid in nature.

A 34-year-old, multiparous woman had been admitted to a rural clinic with the complaint of postcoital bleeding. Due to the detection of a cervical mass, she has been referred to our hospital. We saw a minimally ulcerated mass lesion 3 cm in diameter at the upper ectocervix. On the basis of these clinical findings we performed a colposcopy directed biopsy which resulted as a malignant epithelial tumor. The tumor was staged as 1B1 according to International Federation of Gynecologists and Obstetricians staging system. We performed radical hysterectomy, pelvic-para-aortic lymphadenectomy, bilateral salpingectomy, and ovarian transposition (Figure 1 shows the pathology specimen). The final pathology result revealed mucoepidermoid carcinoma of uterine cervix, grade 2, with intact vaginal surgical border and parametria (Figures 2(a) and 2(b) show histopathologic images with haematoxylin and eosin staining and periodic acid-Schiff staining). There was no lymphovascular space invasion and no metastasis to dissected 43 lymph nodes. On the follow-up we decided chemoradiation therapy on basis of the poor differantiated nature of tumor at our gynecological oncology council. The patient achieved her chemoradiation therapy successfully. After the radiation therapy she had radiation cystitis; she was treated for that and now 12 months after the surgery she is alive with no complaints and no recurrent disease.

## 3. Case Report 2

**Figure 3:**
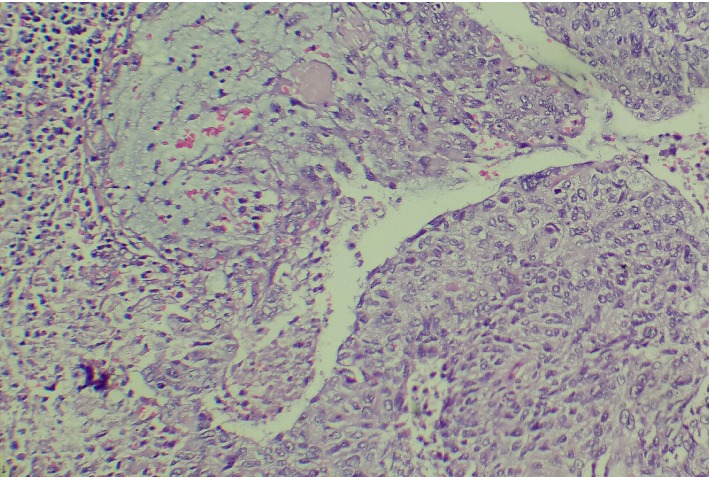
Histopathologic evaluation, H&Ex40: squamous component of tumor cells with intracellular mucin; additionally tumor cells also contain mucin to some extent.

**Figure 4:**
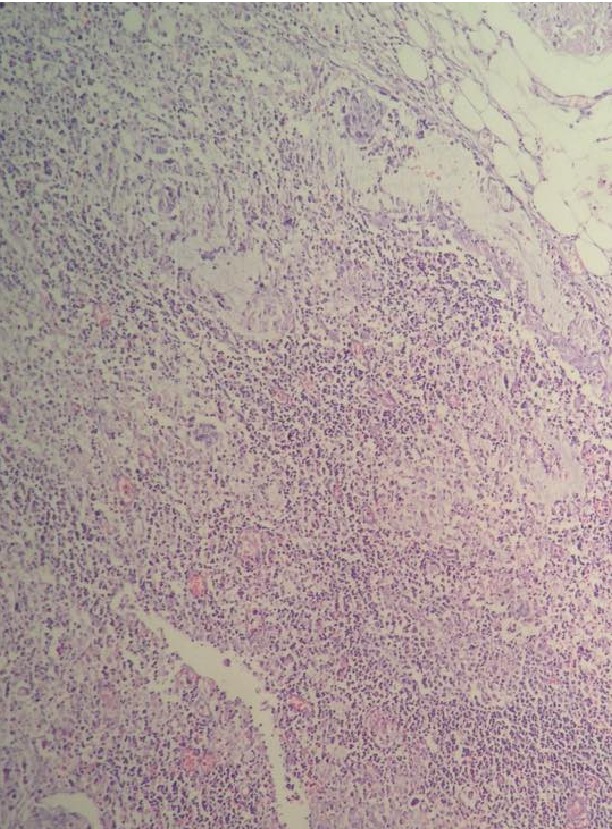
Histopathologic evaluation, H&Ex20: metastatic obturator lymph node with intracellular mucin.

A 63-year-old, multiparous woman was admitted to our outpatient clinic with the complaint of vaginal bleeding. During the pelvic examination we detected a cervical mass 3.5 cm in diameter and took a biopsy from the lesion. Biopsy result was malignant epithelial tumor. She was staged as 1B1 cervical carcinoma. We performed radical hysterectomy, bilateral salpingo-oophorectomy, and pelvic-para-aortic lymphadenectomy. Pathology result defined mucoepidermoid carcinoma of uterine cervix (Figure 3 shows the histopathologic evaluation). Parametria and vaginal border was tumor-free; nevertheless, a lymphatic metastasis was detected at one obturator lymph node out of total of 34 lymph nodes. We detected mucin stained areas during the histopathologic evaluation of metastatic lymph node (Figure 4). Postoperatively she received chemoradiation therapy successfully and now 6 months after the surgery she is without any recurrent disease.

## 4. Discussion

Mucoepidermoid carcinoma of uterine cervix is a malignant tumor and resembles the synonymous tumor of salivary glands. Although the morphology of them is similar, MEC of uterine cervix is not seen as frequent as its counterpart in salivary glands and it has been reported as case series in the literature [3]. While the data are showing some controversies on the classification of MEC, it is grouped as other epithelial tumors of cervix. The unique diagnostic criteria for MEC are the predominance of epidermoid cells, scattered or clumped intermediate cells, and cells containing intracytoplasmic mucin without any glandular differentiation [1]. Thus MECs typically contain three types of cells: squamous cells, mucous cells which have a positive PAS reaction by staining with mucicarmine or Alcian blue, and intermediate cells which are mostly abundant and ranging from small basal cells with basophilic cytoplasm to larger cells which commonly form clusters with eosinophilic cytoplasm [1, 4].

MECs of cervix should be distinguished from endometrial MECs by immunohistochemistry; while anti-CEA is positive and anti-vimentin is negative for cervical MEC, endometrial MEC is negative for anti-CEA and positive for anti-vimentin [5]. We did not perform immunohistochemistry on these patients because the endometrium was fully normal. During the histopathological evaluation both squamous and mucinous components were seen. Squamous components were like nonkeratinized large cells in morphology and mucinous component was in signet and goblet cell nature. Nevertheless we did not find any gland formation. Cervical MECs are distinguished from mucin producing cells by the presence of three cell types and also from adenosquamous carcinomas by the absence of glandular formation [4].

Human Papilloma Virus (HPV) is the main reason of cervical carcinoma. HPV is detected in 99.7% of cervical squamous carcinomas worldwide [6]. Lai et al. [7] analyzed adeno-adenosquamous carcinomas and found HPV 18 positivity for 51.5% of patients and HPV 16 positivity for 36.2% of patients. They found age >50 years, FIGO stage III-IV disease, and HPV 16 negativity as poor prognostic factors and predictors of relapse. We have not evaluated HPV status of our patients.

The degree of mucin production is increased in adenocarcinomas and adenosquamous cancers of cervix whereas it is decreased in squamous cell carcinomas of cervix [8]. Mucin has a value for the prediction of clinical surveillance. Moreover it has been previously reported that mucin producing tumors have an increased potential for metastasizing to the regional lymph nodes [8–10]. Additionally Buckley and Fox [11] recommended that all squamous cell carcinomas of cervix should be stained with specific mucin stains for the prediction of tumor behaviour. Hale et al. [12] analyzed 235 cervical carcinoma patients with stage IB/IIA and found that mucin containing tumors have a higher incidence of lymph node metastasis. Additionally they stated lymph node metastasis as an independent prognostic factor. Ireland et al. [10] also defined a more aggressive nature for intracellular mucin containing cervical carcinomas. The first patient was without any lymphatic metastasis; however the second patient was with lymph node involvement. By the way, an aggressive method of adjuvant therapy and different treatment strategies should be administered to these patients in order to protect early metastasis.

The clinical differentiation of MEC and squamous cell carcinoma of cervix is noteworthy because MECs are commonly more aggressive and generally have a higher potential for metastasizing to lymph nodes than non-mucin-secreting tumors [2, 12]. Thelmo et al. [2] followed up 15 patients for 2 to 15 years and three patients died in the first year. They were having lymph node and vascular metastasis. Reich and Tamussino [5] had stated a case of mucoepidermoid carcinoma with gross omental metastasis. Kim et al. [3] also stated an aggressive mucoepidermoid carcinoma of cervix which was staged as 1B1 without any minor or major risk factors and lymph node metastasis. They did not prefer an adjuvant therapy; however the tumor recurred in four months at the vaginal stump and the patient died 19 months after surgery. We planned chemoradiation combined regimen for our patients because of the poorly differentiated nature of tumor and its aggressive behavior. On the other hand the treatment schedule for mucoepidermoid carcinoma of cervix is not standardised; case reports and previous experiences shape the follow-up choice of clinician.

In conclusion MECs are aggressive tumors and have a predilection for metastasizing to lymph nodes. Recurrence may also occur in patients without any risk factors. For this reason adjuvant chemoradiotherapy should be liberally performed on women with MEC even without any risk factors on the basis of aggressive biologic behaviour of the tumor.
